# Research on developing environmental ethics in pharmacists’ activities

**DOI:** 10.1186/s12940-021-00739-6

**Published:** 2021-05-05

**Authors:** Alexandra Toma, Ofelia Crişan

**Affiliations:** Department of Pharmaceutical Legislation and Management, Faculty of Pharmacy, “Iuliu Haţieganu” University of Medicine and Pharmacy, 12 Ion Creangă Street, Cluj-Napoca, Romania

**Keywords:** Pharmacists, Codes of conduct, Ethical principles, Environmental values, Environmental ethics

## Abstract

**Background:**

Authors of various backgrounds are preoccupied with the meaning of environmental ethics, which refers to specific values, norms, attitudes, and practices with respect to all beings and elements of nature. Many international organizations have adopted important documents proclaiming the ethical obligation to protect the environment in all professional activities. At the national level, professional associations should include and develop this obligation in their codes of conduct. This study aimed to analyze and compare elements of environmental protection included in codes of conduct for pharmacists, to find ways to integrate environmental ethics into their activities.

**Methods:**

Seven codes of conduct for pharmacists were analyzed using the comparative method and methods of legal interpretation. Based on acknowledged values and principles of environmental ethics, a framework model for developing environmental protection in pharmacists’ activities was created via an iterative process of reflection and discussion.

**Results:**

Explicit provisions for environmental ethics were found in codes in force in three countries and in a Romanian project. These provisions refer, in general, to an ethical duty of environmental protection in pharmacists’ activities. Regarding the other codes, the study could only interpret stipulations of public health ethics to deduce an ethical environmental obligation. Considering the need for developing such provisions, an ethical framework was proposed as a model for professional associations of pharmacists.

**Conclusions:**

The studied codes demonstrate a preoccupation of the professional associations with environmental protection in pharmacists’ activities, with different degrees of interest in developing environmental ethics. To this end, the codes of conduct for pharmacists should include environmental values, principles, and ethics guidelines. Those guidelines should indicate the ethical attitude in relation to the environment for each activity. Further research is needed to stimulate, shape, and develop an environmental ethical behavior in pharmacists’ practice.

## Background

Various documents adopted by international organizations proclaim the importance of environmental protection as an ethical obligation in healthcare activities. The World Medical Association (WMA) recommended in the Declaration of Helsinki, as early as 1975, to use caution in medical research that might affect the environment [1], introducing in 2013 the principle of preventive action regarding environmental risks [2]. These principles were also proclaimed in the Universal Declaration on Bioethics and Human Rights, which supported taking into consideration the environmental impact of healthcare activities [3]. More recently, they were reiterated and further developed in the Declaration of Ethical Principles in relation to Climate Change, including encouragement of environmental education, evaluation and management of environmental risk posed by the new technologies resulting from scientific research, ensuring their compatibility with environmental sustainability, public participation in the spirit of equity and justice, and solidarity in protecting the planet [4]. In the European Union (EU), the solidarity-related rights of citizens have an environmental quality protection component, included in the Charter of Fundamental Rights of the EU [5]. The increase in the quality of environmental factors should be an important part of any public health policy. For example, the World Health Organization guidelines on ethical issues in public health surveillance establish that states have the ethical obligation to protect public health, including by monitoring and optimizing the environment, which involves community participation and commitment, meaning solidarity and stewardship [6]. It is recognized that ethics are a motivating factor in developing environmental stewardship [7–9], including in a professional context [10].

Consequently, an important method for the development of environmental ethics in healthcare activities is to include specific principles in the codes of conduct of health professionals. For example, the European Federation of Clinical Chemistry and Laboratory Medicine included the principle of environmental caution in the Code of conduct for specialists in this field [11]. Another mode of action is that of the International Pharmaceutical Federation, which proclaimed the principles of environmental responsibility, professional collaboration, and involvement of the public in environmental protection activities in the reference document “Green pharmacy practice: Taking responsibility for the environmental impact of medicines” [12], intended for pharmacists and their professional associations. At the national level, professional associations are responsible for adopting and developing all these principles in their codes of conduct [13, 14]. In fact, since the United Nations Conference on Environment & Development, known as The Earth Summit, which concluded with the Rio Declaration, the international community has relied on public participation and, in Agenda 21, on an important role played by professionals and scientists in environmental protection, including by developing environmental ethics guidelines and codes of conduct [15, 16]. Following the Earth Summit, a movement for establishing the global “values and principles for a sustainable future” was initiated, leading to the Earth Charter, a universal declaration regarding the planet as our most important responsibility [17].

Authors with various backgrounds support the formulation of environmental ethical values, ideals, principles, and standards in codes of conduct for health professionals, to help develop a moral relationship between them and the environment, including by correlation with bioethics and public health ethics [10, 18–21]. Thus, Lee considers that public health ethics is a link between bioethics and environmental ethics, given that it deals with individual health as well as with community and environmental health, solidarity, and justice in these areas [18]. In fact, many authors consider that it is an ethical obligation of health professionals to educate themselves in environmental protection as well as to participate in educating patients and local communities, including by campaigns for the promotion of environmental quality, as a determining factor of public health [18, 19, 21–25]. Rozzi believes that the relationship between human health and the environment should also be viewed through a biocultural ethics lens and that we should appreciate the value of the traditional cultural relationship of various local communities with nature and participate in its conservation [26]. The principle of conservation is analyzed in the context of environmental ethics by Arntzen, who supports its application, along with the principle of sustainability, to preserve the beauty of nature, by avoiding aggressive human action on the environment [27].

Often, through various activities, people harm the beauty of nature or the harmony of the environment in which we live, including the urban environment. Ignoring the impact of human activity on the beauty of nature and environmental harmony is an attitude that contributes to a decrease or deterioration of environmental quality, which entails a reduction of quality of life and, by extension, of human health [9, 27, 28]. In fact, many authors support the need for more intense policy-making participation from professionals concerned with environmental ethics, both personally and through their professional associations [10, 18–22, 25, 26, 29]. Further, these professionals, as moral community models, should participate, individually or through non-governmental organizations, in debates and actions dedicated to environmental cleaning and restoration [18, 21, 30]. At an institutional level, searching for and applying organizational and technological solutions to implement environmental protection principles, such as preventive action, stewardship, and conservation of nature, should be ethical obligations, even internally regulated [8, 10].

Further studies on the boomerang relationship between human activity with a negative environmental impact and human health are required [20, 21, 25, 29], but research to minimize this impact, including through the development of codes of conduct and ethical practices, is a priority. According to The Stanford Encyclopedia of Philosophy “environmental ethics is the discipline in philosophy that studies the moral relationship of human beings to, and also the value and moral status of, the environment and its non-human contents” [31]. Ethical issues, related to the need for philosophical, organizational, and technological changes, can be raised in connection with the environmental “unhealthy impact” of health care [32]. Pharmacists, as health care professionals, are engaged in many activities regarding human drugs, from research and authorization, through manufacturing and distribution, to prescribing and dispensing in pharmacies and hospitals, all those involving important amounts of energy, water, equipment, instruments, active ingredients, packages, cleaning products, and waste [12, 33, 34]. Among the different issues covered by environmental ethics [31], we believe, and through our study propose, that pharmacists should develop their ethical behaviors regarding social responsibility, waste management, beauty and the built environment, sustainability, and climate change. To our knowledge, there are no academic publications providing an environmental ethics framework for pharmacists’ activities. Consequently, given the international context and the need for such research, this study aims to identify and analyze elements of environmental ethics in codes of conduct for pharmacists and to create an ethical framework model for developing environmental protection in pharmacists’ activities.

## Methods

### Comparative study

In the first part of the research, a comparative study of pharmacists’ codes of conduct of seven countries was conducted, to identify elements of environmental ethics. As this study is a continuation of previous research, it includes Romania and another six advanced countries, whose environmental protection laws and practices in pharmacists’ activities were analyzed in previous studies and that were considered models to follow for the development of this field in Romania [13, 14, 35]. Consequently, the codes of conduct adopted by the associations of pharmacists in Australia, Finland, France, Hungary, Spain, Romania, and the United States (US) were studied. These codes were analyzed using the comparative method, which involves an analysis of the terms to be compared and a comparative synthesis, which includes a discussion of similarities and differences between these, based on chosen comparison criteria [36]. The following aspects were used as comparison criteria: the presence of explicit provisions of environmental ethics in the text of the code, the content of these provisions (when they were present), and the content of implicit provisions (when there were no explicit provisions). Their content was interpreted using the literal method, looking at the formulation of the provisions, and the teleological method, looking at their spirit [37].

### Ethical framework

In the second part of the research, we created an ethical framework model for environmental protection, as this is an important social issue to be considered by professional codes of conduct according to Summers et al. [38]. This model could be useful in developing environmental ethics in codes of conduct for pharmacists. For the creation of this model, categories of elements from the moral principles’ framework of Beauchamp and Childress [39] and the ethical analysis framework of Veatch, Haddad, and Last [40] were used. Starting from these, the following categories of elements were included in the framework model: values – defined as good, beneficial things, to be protected or maximized in relation to the environment, principles – defined as general moral rules of action for protecting values, and ethics guidelines – defined as sets of specific ethical rules required to orient professionals in implementing the principles [39, 40]. The decision on which elements to include in the framework model, in each of these categories, was taken via an iterative process of reflection and discussion following the review of current literature.

## Results

Explicit environmental ethics provisions can be found in the Australian Code [41], the Hungarian Code [42], the Spanish Code [43] and a Romanian project [44]. Only implicit environmental ethics provisions can be found in the Finnish Ethical Guidelines [45, 46], the French Code and a new project [47, 48], the US Code [49] and the Romanian Code [50]. All provisions are formulated as general and/or specific ethical obligations, as shown in Table 1.

**Table 1:** Environmental protection in pharmacists’ codes of conduct

Country	Criteria	
	Content of explicit provisions	Content of implicit provisions
Australia	General obligation for pharmacists to protect the environment	
Finland		General obligation for pharmacists and pharmacies to answer the health needs of the patients, community, and society
France		General obligation for pharmacists to act in the interest of patients and public health
Hungary	General obligation for pharmacists to protect the environment	
Romania	General obligation for pharmacists to protect the environment^a^	General obligation for pharmacists to act in the interest of patients and public health
	Specific obligations for pharmacists in pharmacies regarding environmental protection: waste management and patient counseling^a^	
Spain	General obligation for pharmacists to protect the environment	
	Specific obligations for pharmacists regarding environmental protection: waste management and patient counseling	
US		General obligation for pharmacists and pharmacies to answer the health needs of the patients, community, and society

^a^New deontological code project, yet unadopted by the Romanian College of Pharmacists

An initiative for the development of environmental ethical behaviors in pharmacists’ activities should involve the inclusion of environmental protection as an explicitly formulated fundamental principle in their codes of conduct, the design of an adequate ethical framework, and continuous training programs for its implementation. Such an initiative would imply an ethical reflection exercise, within the professional association, for organizing and formulating the text categories to be included in the code, their discussion, adoption, and implementation. A model of such an ethical framework, resulting from our research and reflection, is presented in Table 2.

**Table 2:** Ethical framework for environmental protection in pharmacists’ activities

Values	Principles	Ethics guidelines for:
Life Health Environmental rights: the right to a healthy environment	Principle of stewardship	Protecting the environment by promoting and implementing sustainable practices and climate change mitigating measures
	Principle of caution	Including environmental ethics rules in the drug marketing authorization process
	Principle of preventive action	Preventing the generation of pharmaceutical waste
	Principle of responsibility	Taking responsibility for appropriate pharmaceutical environmental management
	Principle of collaboration	Initiating pharmaceutical environmental projects or adding a pharmaceutical dimension to environmental protection projects
	Principle of justice	Promoting a fair and equitable allocation and use of pharmaceutical resources, including in environmental management
Freedom Safety and security Environmental rights: the right to enforce environmental rights	Principle of public participation	Participating in environmental governance in the pharmaceutical field
Beauty Cultural rights Environmental rights: the right to the beauty of nature and environmental harmony	Principle of environmental preservation	Participating in protecting the beauty of natural areas and the harmony of the urban environment

## Discussion

Public health is strongly determined by the quality of environmental factors [6, 18]. Therefore, all the implicit provisions of the studied codes of conduct were interpreted as involving pharmacists’ moral obligation to act for the protection of these factors. This interpretation is in full agreement with the French drug waste management system [51], with the role Finland and US attribute to pharmacies in collecting expired drugs from the population, an activity performed by various public authorities as well [52, 53], and with the important concern for the impact of drugs on the environment in Northern European countries in general [54, 55].

The Spanish Code and the Hungarian Code are in accordance, respectively, with the two national drug waste management systems [56, 57]. The Romanian project would establish new environmental ethical obligations for pharmacists, reflecting their will to progress [14, 44, 58]. The current Romanian Code foresees that pharmacists should play an active role in fighting the scourges of the modern world [50], pollution being one of them [20, 21, 29]. Unfortunately, no initiative of Romanian pharmacists has been identified in this regard. The environmental ethical education is at its beginning in our schools of pharmacy, and we are pioneers, together with our students. We, as pharmacists and teachers, follow the Earth Charter by integrating into our lectures and seminars “knowledge, values, and skills needed for a sustainable way of life” [17], which are discussed through various team projects.

The Australian Code stands out among all the other codes through the care for “equitable and sustainable use of healthcare resources in an environmentally responsible manner,” through the promotion of “environmental responsibility and accountability” in all pharmacists’ activities [41]. We completely agree with these formulations and admire the quality of the Code and work ethic of the Pharmaceutical Society of Australia. There is a certain resemblance with the US Code, which establishes the obligation of “seeking justice in the distribution of health resources,” by being “fair and equitable, balancing the needs of patients and society” [49]. In so far as that pharmacists’ activities also involve the management of environmental resources and risks for health and the support of activities allowing poor or disadvantaged communities to gain access to higher-quality environmental factors [10, 18–20, 29], those provisions were interpreted as including an environmental ethical obligation.

The analysis of the 7 codes of conduct shows various degrees of preoccupation of pharmacists’ professional associations with ethics in environmental protection. In comparison with the matters covered by the concept of environmental ethics [31], the main issues addressed in the studied codes of conduct are social responsibility and waste management, with less focus on beauty and the built environment, sustainability, and climate change. Therefore, we proposed a more consistent approach to developing environmental ethics in pharmacists’ activities, by creating an ethical framework model.

The first values to be protected and included in the framework model were life, health, and the right to a healthy environment, because they are intricately connected, as has already been proclaimed internationally since the Rio Declaration [15]. But rights have corresponding duties, such as to avoid any environmental damage and to respect other people’s environmental rights, according to the Earth Charter. This document also talks about the community of life that we must respect and care for “with understanding, compassion, and love” [17]. In addition, the United Nations Environment Program currently maintains that it is no longer a matter of just the life and health of humans but of the whole planet that we must take care of because “environmental degradation impacts on the enjoyment of human rights” [59]. In fact, other authors posit that we must have “a truly ethical relationship with the planet that we inhabit” [22] or “an approach to ethics [ …] that takes human health and the environment into account” [20], a balanced approach that is also the aim of the present research. Thus, six principles by which these values can be defended were identified.

The principle of stewardship would require the elaboration of ethics guidelines for researching and implementing green institutional and technological alternatives (e.g., sustainable buildings, renewable energy, saving water) and for developing good green practices (e.g., managing pharmaceutical waste, recycling drug packages, saving paper), as moral obligations of sustainability and climate change mitigation [31]. The pharmacists’ involvement in monitoring and restoring environmental quality, including in the context of solidarity with the communities affected by its degradation (e.g., local actions of environmental cleaning, planting trees, providing environmental education), would be extremely useful in the organic process for developing guidelines. These modalities were formulated by adapting stewardship actions [7] promoted at a global scale [6, 12, 17].

The principle of caution refers, in the context of the framework model, to showing prudence in marketing drugs with a high risk to environmental quality. Its application would require ethics guidelines including stakeholders’ obligations to publicize the results of environmental risk assessments for drugs, consider these results when evaluating their risk-benefit balance, and implement specific measures for minimizing risks. Given that there is insufficient evidence of the impact that the presence of drugs in the environment may have on human health in the long term, a wise, cautious approach is necessary [60]. These modalities would also require legal regulation, as we have advocated in previous studies [13, 14, 35], but these are also moral obligations of those responsible for marketing the new drugs and health technologies, which can be addressed specifically in ethics guidelines for pharmacists.

The principle of preventive action would require ethics guidelines for the rational prescribing and dispensing of drugs, including depending on their environmental impact, for example considering the results of already published evaluations by competent authorities, such as that in the Stockholm Region [61]. Moreover, guidelines for the inclusion of pharmacists in patient therapy management teams might contribute to both rational drug use and drug waste reduction, as demonstrated by some promoted British projects [62, 63].

The principle of responsibility for the environmental impact of health care activities is proclaimed by many authors [12, 32–34]. Ethics guidelines would be useful for involving pharmacists in drug waste management systems, counseling the public about good green practices, and disseminating information, following the Spanish ethical models [56, 64, 65]. In this context, however, we strongly maintain that the actual point of environmental ethics should be to provide moral grounds not only for social policies [31], but also for plans and strategies for developing mindfulness about non-human inhabitants of the Earth.

The principle of collaboration is already applied in some European countries where non-profit organizations have been created for the management of drug waste collected from the population, within which different types of pharmaceutical stakeholders (manufacturers, wholesale and retail distributors, professional associations) collaborate with patients and public authorities [51, 56, 57]. It would be particularly useful for the development of ethics guidelines the participation of pharmacists in discussions, workshops, common programs, and projects, alongside other specialists, institutions, authorities, or communities. Also, ethics of sustainability and climate change could be applied in practice by pharmacists through collaboration with architects, designers, and managers, to reduce energy consumption, thus the carbon footprint, or to select fair trade providers and eco-friendly equipment, instruments, products etc. [12, 32–34, 65] in pharmacies, hospitals, and other pharmaceutical units.

The principle of justice would need to be codified for an equitable allocation between manufacturers and distributors not only of benefits related to the marketed drugs, but also of risks and costs related to the disposal of expired or unused drugs collected from the population [35]. Further, this principle would apply through the manifestation of solidarity, through non-discrimination and facilitation of equitable access to pharmaceutical care specifically for populations at risk or already affected by the degradation of environmental quality, which are primarily vulnerable, poor communities [8]. Simultaneously, as Cabrera et al. show, participating in the search and implementation of solutions to minimize the negative impact of environmental quality changes on society is crucial and can be achieved by contributing to policymaking [29], including by political mobilization [22], for better environmental governance [59]. In this context, we agree that an important ethical task of societies is “to fashion institutions and forms of life that promote health in ways that recognize the claims of sustainability and justice” [10]. Recently, the concept of “planetary justice” was proposed to be used instead of “planetary ethics,” as a field of research related to the interests of humans, non-human animals, and non-sentient nature, including current and future generations, intrinsic and instrumental reasons, and existing and required mechanisms for the protection of the planet, with important normative implications and moral imperatives [66]. The link between socio-economic justice and environmental protection has already been elegantly promoted in the Earth Charter as a pylon for the future of the planet [17].

Another fundamental value in the framework model is freedom, as individuals need to be free to make informed decisions, travel, discover the planet, and express themselves on environmental issues. With those rights comes the people’s duty to protect the planet as their home, the quality of its natural elements and its biodiversity, the integrity of “Earth’s life support systems” [17]. This is imperative for the safety of humans and other beings, especially with environmental crimes having become an emerging threat that requires the development of environmental security actions [59]. Therefore, safety and security are extremely important ethical values for environmental protection [8, 67]. In addition to the actions taken by enforcement agencies, individuals have the right to enforce environmental rights, by applying the principle of public participation. In this context, an aspirational code [38] would work better for pharmacists, as liberal professionals, and it should provide ethics guidelines for participating, individually or within a professional association, in environmental governance. However, the code should include prescriptive statements [38] on public advocacy, useful for pharmacists involved in monitoring the environmental obligations of pharmaceutical authorities (e.g., requesting the fulfillment of environmental obligations by the drug agency, including the publication of information of public interest), consulting for regulations and policies related to the environment, or defending environmental rights in court and claiming compensation for any damage suffered. In fact, the importance of public participation in environmental stewardship, based on intrinsic and extrinsic motivations, was brilliantly documented, and argued by Bennett et al. [7], and the examples of community involvement in various countries can represent models of conduct to be followed in the pharmaceutical field as well. The organic process for developing ethics guidelines or codes of conduct would also benefit from the involvement of pharmacists in such community actions, as already testified in practice [12, 33], having a great impact on transforming the way of thinking and acting, not only as pharmacists, but also as individuals.

Finally, the beauty of nature is a basic value for people’s relationship with the environment, being related especially to cultural human rights [9, 26, 28, 59]. Some authors even support the right to beauty, which involves the right to not only enjoy the beauty of nature but also to live and work in a harmonious environment that may include urban beauty [67], the built environment being the main space of human life [31]. Also, the Earth Charter proclaims the duties of protecting “places of cultural and spiritual significance” and securing the beauty of the planet for next generations [17]. This immeasurable value of beauty can be protected in the pharmaceutical field by including the principle of preservation in codes of conduct, along with ethics guidelines referring, for example, to the design and organization of professional pharmaceutical premises in harmony with the environment (nature, architecture), while considering aesthetic criteria. Ideally, these criteria should be used in urban planning and development policies, on account of the right of communities to beauty [67], to improve social interaction [31], thus quality of life. In this context, ethics guidelines would be useful to orient and help pharmacists in promoting and supporting the adoption by public authorities of regulations for protecting nature (e.g., development of green areas, parks, botanical gardens) and the harmony of the urban environment, in particular historically or artistically protected areas (e.g., interdiction of discordant buildings, restoration and maintenance of pharmacy history museums, pharmacies with historical or artistic value). Some authors show that considering the aesthetic criteria in environmental governance can stimulate ethical behavior and commitment to environmental protection [9].

The present research agrees with the results of the studies of all these fellow authors, as we found our reflections and ideals expressed in their writings. Our framework model is not exhaustive, so other elements could be added depending on what those interested consider being relevant and necessary. Further research is needed on how to stimulate, shape, and develop an environmental ethical behavior in pharmacists’ practice. Our experience, as pharmacists, teachers, and researchers, tells us that attractive strategies of communication, flexible training programs, encouraging new ideas, leading by example, and providing successful models from other communities, professionals or facilities are the best ways for a quick start of a process of accepting and adopting a new approach to professional actions in relation to environmental protection.

## Conclusions

Overall, given their wide access by the public, pharmacists’ activities can have an important impact on the population’s health and education, including regarding environmental protection. The participation and commitment of professional associations are required for the development of environmental ethics in pharmacists’ activities. A code of conduct for pharmacists should include specific values, principles, and ethics guidelines to contribute to reaching this goal. Environmental values are connected to fundamental human rights and can be defended by applying internationally recognized principles of action. Ethics guidelines should indicate the best ethical behavior for each activity in relation to the environment. Our study is the first to compare environmental ethics provisions in codes of conduct for pharmacists and to provide a framework model for developing such provisions. Consequently, its importance and relevance reside in highlighting different approaches of environmental ethics and providing a basis for discussing the need to develop this matter among professional pharmacists.

## Data Availability

Not applicable.

## References

[1] World Medical Association (1975). Declaration of Helsinki – Recommendations guiding medical doctors in biomedical research involving human subjects.

[2] World Medical Association (2013). WMA Declaration of Helsinki – Ethical principles for medical research involving human subjects.

[3] United Nations Educational, Scientific and Cultural Organization (UNESCO) (2005). Universal Declaration on Bioethics and Human Rights.

[4] United Nations Educational, Scientific and Cultural Organization (UNESCO) (2017). Declaration of Ethical Principles in relation to Climate Change.

[5] European Union (2016). Charter of fundamental rights of the European Union. OJEU..

[6] World Health Organization (2017). WHO guidelines on ethical issues in public health surveillance.

[7] Bennett NJ, Whitty TS, Finkbeiner E, Pittman J, Bassett H, Gelcich S, et al. Environmental stewardship: a conceptual review and analytical framework. Environ Manag. 2018;61(4):597–614. 10.1007/s00267-017-0993-2. 10.1007/s00267-017-0993-2 PMC5849669 29387947

[8] Børsen T, Nielsen SN (2017). Applying an ethical judgment model to the case of DDT. HYLE – Int J Philosophy Chem.

[9] Richardson BJ, Barritt E, Bowman M (2019). Beauty: a lingua franca for environmental law?. Transnational Environ Law.

[10] Dwyer J (2009). How to connect bioethics and environmental ethics: health, sustainability, and justice. Bioethics..

[11] McMurray J, Zerah S, Hallworth M, Koeller U, Blaton V, Tzatchev K, et al. The European Register of Specialists in Clinical Chemistry and Laboratory Medicine: Code of Conduct, Version 2–2008. Clin Chem Lab Med. 2009;47:372–5. 10.1515/cclm.2009.082. 10.1515/cclm.2009.082 19676151

[12] International Pharmaceutical Federation (FIP) (2015). Green pharmacy practice. Taking responsibility for the environmental impact of medicines.

[13] Toma A, Crişan O (2021). Regulations on green pharmacy in European countries – a comparative study. Farmacia.

[14] Toma A, Crișan O (2019). Environmental protection in the codes of ethics for pharmacists. Med Pharm Rep.

[15] United Nations General Assembly (1992). Report of the United Nations Conference on Environment & Development. Annex I Rio Declaration on Environment and Development.

[16] United Nations Conference on Environment & Development (1992). Agenda 21.

[17] Earth Charter Initiative (2001). The Earth Charter.

[18] Lee LM (2017). A bridge back to the future: public health ethics, bioethics, and environmental ethics. Am J Bioeth.

[19] Jennings V, Yun J, Larson L (2016). Finding common ground: environmental ethics, social justice, and a sustainable path for nature-based health promotion. Healthcare..

[20] Resnik DB (2009). Human health and the environment: in harmony or in conflict?. Health Care Anal.

[21] Valles SA (2015). Bioethics and the framing of climate change’s health risks. Bioethics..

[22] Dunk JH, Jones DS, Capon A, Anderson WH (2019). Human health on an ailing planet – historical perspectives on our future. N Engl J Med.

[23] Nneji B, Nneji C (2019). Complementing public health education with environmental ethics education in the face of challenges of climate change. Am J Health Educ.

[24] Li AM (2015). Ecological determinants of health: food and environment on human health. Environ Sci Pollut Res Int.

[25] Shriver AJ, Cabrera LY, Illes J (2017). Environmental neuroethics: bridging environmental ethics and mental health. Am J Bioeth.

[26] Rozzi R (2016). Global bioethics and biocultural ethics. Cuad Bioet.

[27] Arntzen S (1999). Natural beauty, ethics and conceptions of nature. Filozofski vestnik.

[28] Rolston H, Berleant A (2002). From beauty to duty: aesthetics of nature and environmental ethics. Environment and the arts: perspectives on environmental aesthetic.

[29] Cabrera LV, Tesluk J, Chakraborti M, Matthews R, Illes J (2016). Brain matters: from environmental ethics to environmental neuroethics. Environ Health.

[30] Alblas E (2018). Conflicting goals and mixed rationales: a closer look at the objectives of EU environmental law in light of the Anthropocene. Rev Eur Comp Int Environ Law.

[31] Brennan A, Yeuk-Sze L, Zalta EN (2020). Environmental ethics. The Stanford Encyclopedia of Philosophy.

[32] Jameton A (2009). Medicine’s role in mitigating the effects of climate change. Virtual Mentor.

[33] Ientile G (2020). The carbon footprint in the room: ‘going green’ in the pharmacy. Drug Topics.

[34] Health Care Without Harm (2019). Health care’s climate footprint.

[35] Toma A, Crișan O (2020). Activities related to human medicines in Romania: legal environmental protection issues. Environ Sci Pol.

[36] Constantinesco LJ (1998). Treaty of comparative law, tome 2: comparative method.

[37] Delnoy P (2008). Éléments de méthodologie juridique: 1. Méthodologie de l'interprétation juridique; 2. Méthodologie de l'application du droit. 3e éd.

[38] Summers C, Soskolne CL, Gotlieb C, Fawcett E, McClusky P (1995). Do scientific and scholarly codes of ethics take social issues into account?. Account Res.

[39] Beauchamp T, Childress J (2019). Principles of biomedical ethics.

[40] Veatch RM, Haddad A, Last EJ (2017). Case studies in pharmacy ethics.

[41] Pharmaceutical Society of Australia (2017). Code of ethics for pharmacists.

[42] Hungarian Chamber of Pharmacists (2020). Code of ethics.

[43] Consejo General de Colegios Oficiales de Farmacéuticos (2018). Código de deontología de la profesión farmacéutica.

[44] Romanian College of Pharmacists (2015). Project of the pharmacist’ deontological code.

[45] Finnish Pharmaceutical Association: Ethical guidelines for pharmacists. https://www.farmasialiitto.fi/tietoa-farmasian-alasta/farmasian-ammattilaisen-etiikka/farmasistin-eettiset-ohjeet.html#a282d306. Accessed 5 Jan 2021.

[46] Finnish Pharmaceutical Association (2019). Ethical guidelines for pharmacy operations.

[47] Ordre National des Pharmaciens (2009). Code de déontologie des pharmaciens.

[48] Ordre National des Pharmaciens (2018). Projet de code de déontologie des pharmaciens et autres dispositions à insérer dans le code de la santé publique.

[49] American Pharmacists Association (1994). Code of ethics for pharmacists.

[50] Romanian College of Pharmacists (2009). Decision no. 2/2009 regarding the approval of the Statute of the Romanian College of Pharmacists and of the pharmacist’ Deontological Code. Official J Romania.

[51] Cyclamed (2018). Thinking Cyclamed.

[52] Finnish Medicines Agency (FIMEA) (2020). How should medicines be disposed of?.

[53] US Food and Drug Administration (2020). Drug disposal. Drug take back locations.

[54] Lääkkeetön Itämeri (2020). Drug-free Baltic Sea.

[55] Pharma Industry Finland: Medicines and the environment. https://www.pif.fi/responsibility/medicines-and-the-environment.html. Accessed 5 Jan 2021.

[56] SIGRE (2021). Stakeholders.

[57] Recyclomed (2015). Our pharmaceutical waste collection activity.

[58] Crişan O, Iacob S (2018). Romanian code of pharmaceutical deontology – a new conception. Farmacia..

[59] UN Environment Programme (2019). Environmental governance.

[60] Janusinfo Region Stockholm (2018). Impact of pharmaceuticals on the environment.

[61] Janusinfo Region Stockholm (2020). Pharmaceuticals and environment.

[62] National Institute for Health and Care Excellence (NICE) (2014). Pharmacist-led repeat prescription management: ensuring appropriate prescribing and reducing wastage.

[63] National Institute for Health and Care Excellence (NICE) (2018). Utilising the skills of the clinical pharmacist within the MDT for improved medicines optimisation.

[64] Smart Hospital (2017). European Project Life+: Results obtained.

[65] SIGRE (2021). Ethics and transparency.

[66] Hickey C, Robeyns I (2020). Planetary justice: what can we learn from ethics and political philosophy?. Earth System Governance.

[67] Harvey A, Julian C (2015). A community right to beauty: giving communities the power to shape, enhance and create beautiful places, developments and spaces.

